# Multi-level coding-recoding by ultrafast phase transition on Ge_2_Sb_2_Te_5_ thin films

**DOI:** 10.1038/s41598-018-23360-z

**Published:** 2018-03-21

**Authors:** Shuai Wen, Yun Meng, Minghui Jiang, Yang Wang

**Affiliations:** 1 Key Laboratory of High Power Laser Materials, Shanghai Institute of Optics and Fine Mechanics, Chinese Academy of Sciences, Shanghai, 201800 China; 2 University of the Chinese Academy of Sciences, 100080 Beijing, China

## Abstract

Quickly switching among different states (levels) is crucial for reconfigurable metamaterials and devices. In this study, the dynamics of establishment and transformation of five amorphous or near-amorphous intermediate states with obvious optical contrasts on Ge_2_Sb_2_Te_5_ phase-change thin films driven by ultrashort laser pulses were investigated using real-time reflectivity measurements. The reversible coding-recoding among the five optical levels was realized by using single-shot picosecond laser pulses with designed fluences. The optical constants, crystalline states and surface morphologies before and after ultrafast multi-level coding were also compared and analyzed. These results may lay a foundation for the further design and application of dynamically reconfigurable optical/photonic devices.

## Introduction

External-field-driven refractive-index-changing phase-transition has been proven to be an effective way to construct switchable and reconfigurable optical/photonic metamaterials, components, and devices^1,2^.

The phase-changeable chalcogenide glasses represented by Germanium-Antimony-Telluride (GeSbTe), widely used as non-volatile binary coding media in optical and electrical data storage, can offer a uniquely flexible platform for the realization of these kinds of materials and devices, owing to their several extraordinary properties^3–5^: (1) obvious distinctions of optical and electrical properties such as refractive index and electrical conductivity between crystalline and amorphous phases; (2) fast and reversible transformation between the two phases driven by optical, electrical, and thermal energy; and (3) sufficient thermal stability of both the metastable amorphous phase and energetically favorable, stable crystalline phase.

Furthermore, metastable partially crystallized states can be created by carefully controlling the driving energy per unit area and time to achieve multi-level coding^6^, which will enrich the functions of phase-change-type reconfigurable optical/photonic metamaterials and devices.

Conventionally, the crystallization process is often achieved by a moderate and long laser pulse, and is not favored by using ultrashort laser pulses^7,8^ because of the minimum time required for crystalline nucleation to form and grow^9,10^. However, studies showed that by carefully modifying the way heat is extracted from the substrate^11,12^, cycling the materials between crystalline and amorphous phases with ultrashort pulses can be fulfilled^13,14^. What’s more, ultrashort laser pulse driven multi-level crystallization has been realized for all-photonic multi-level memory^15^, grayscale image (hologram) recording^16,17^, color display^18^, cognitive computing^19^, and so on.

Although multi-level coding with ultrafast lasers on a phase change material has attracted considerable attention and been widely investigated, most of the studies focus on cumulative switching (only one-way crystallization process in most cases), which means repeatedly exposing the same area of the phase change thin film to quasi-continuous ultrafast laser pulses and modulating the degree of crystallization by controlling the accumulation of heat^16,18^. In contrast, reversible multi-level phase change modulation using a single-shot ultrashort pulse has been seldom reported, yet with this method, the time duration required for reaching a certain state can be greatly reduced and the attached thermal diffusion effect can be constrained. Quickly switching among different states in a localized area is crucial for the construction of dynamically reconfigurable units.

Accordingly, in this study, by applying single-shot 30-ps laser pulses with designed fluences, five different amorphous or near-amorphous intermediate optical states (levels) were established and transformed between each other on the Ge_2_Sb_2_Te_5_ phase-change thin film. The time-resolved dynamic coding-recoding of the reflectivity contrast was studied in detail with a pump-probe system. Those five coded optical states were characterized comprehensively by spectral ellipsometer measurements, XRD analysises and surface morphology observations. To the best of our knowledge, the ultrafast reversible phase transition processes among different amorphous or near-amorphous intermediate states were reported for the first time.

## Results and Discussion

### Establishment of different optical states

**Figure 1:**
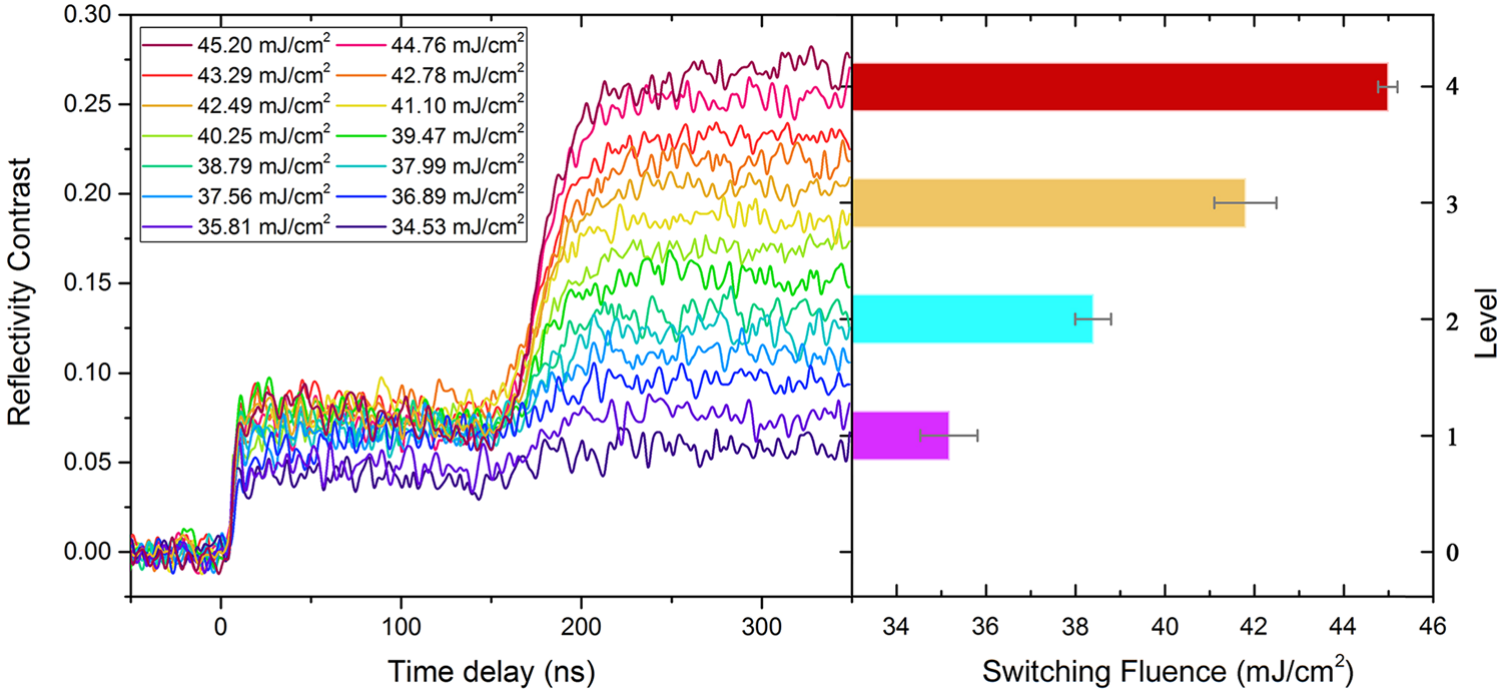
Real-time reflectivity contrast evolution during the establishment process of four optical states starting from as-deposited Ge_2_Sb_2_Te_5_ thin films and corresponding laser fluence ranges.

As shown in Fig. 1, by using single-shot ps-laser pulses with well-chosen fluences ranging from 34.53–45.20 mJ/cm^2^, a dozen different optical states were clearly established with varieties of reflectivity contrasts (RCs), which are calculated by $(R-{R}_{am})/{R}_{am}$, where R is the final reflectivity after irradiation of ps-laser pulses and R_am_ represents the initial reflectivity of the as-deposited amorphous thin film. The initial zero RC (level 0) and various final RCs after irradiation reflect the difference in the crystallization fraction of the phase change thin films^20^. As we know, the reliable confidence interval is the key factor that defines and identifies different levels^15^. To ensure the operability, only four non-zero states (level 1–level 4) are chosen and the feature RCs of the four states are 6.5%, 13%, 19.5%, and 26%, respectively, where the floating range is ±1%. Obviously, the separation between every contiguous state (approximately 6–7% in the RC) is enough to tell them apart. Besides the separations in the RC (>5%), those in the fluence range between every state are high enough (>3 mJ/cm^2^ for the mean values). The corresponding laser fluence ranges for the establishment of each non-zero states are 34.53–35.81, 37.99–38.79, 41.1–42.49, and 44.76–45.2 mJ/cm^2^, respectively. When the pulse fluence is higher than 46 mJ/cm^2^, the final RC will drop down obviously (not shown here) due to the surface ablation. These states are not included in the above-mentioned non-zero states although they may have similar RCs.

From the dynamics of the RC, we can see that the crystallization process (corresponding to the curves in which the final RC is higher than the starting one) from the as-deposited amorphous state is a multi-stage process. Upon laser pumping, the RC sharply increases at first (within ~10 ns), then slowly decreases to a metastable state (within ~150 ns) and at last quickly increases to the final stable state (within ~50 ns). The time duration of the entire multi-stage process was approximately 210 ns, and did not obviously change with the fluences.

In fact, similar multi-stage transients have been commonly observed in GeSb and Ge thin films upon picosecond and nanosecond laser pulse irradiation^21,22^. A recalescence process is thought to be involved in this kind of multi-stage crystallization. With the irradiation of the single-shot ps-pulse, the as-deposited amorphous thin film is heated beyond the crystallization threshold, and due to the ultrashort heating time, a small number of nuclei and crystallites generate. This partial solid-state crystallization process^14^ may account for the sharp increase in the RC during the first stage. Then, with the heat released from crystallization, the temperature at the film surface may increase beyond the melting point, which was evidenced by the gradual decrease in the RC. However, during the subsequent temperature decrease and solidification processes, the released enthalpy lowers the cooling rate, frustrates the amorphization attempt and promotes the nucleation and growth of the crystalline phase^23^. This recalescence phenomenon may result in the second increase in the RC.

### Transformation among different optical states

**Figure 2:**
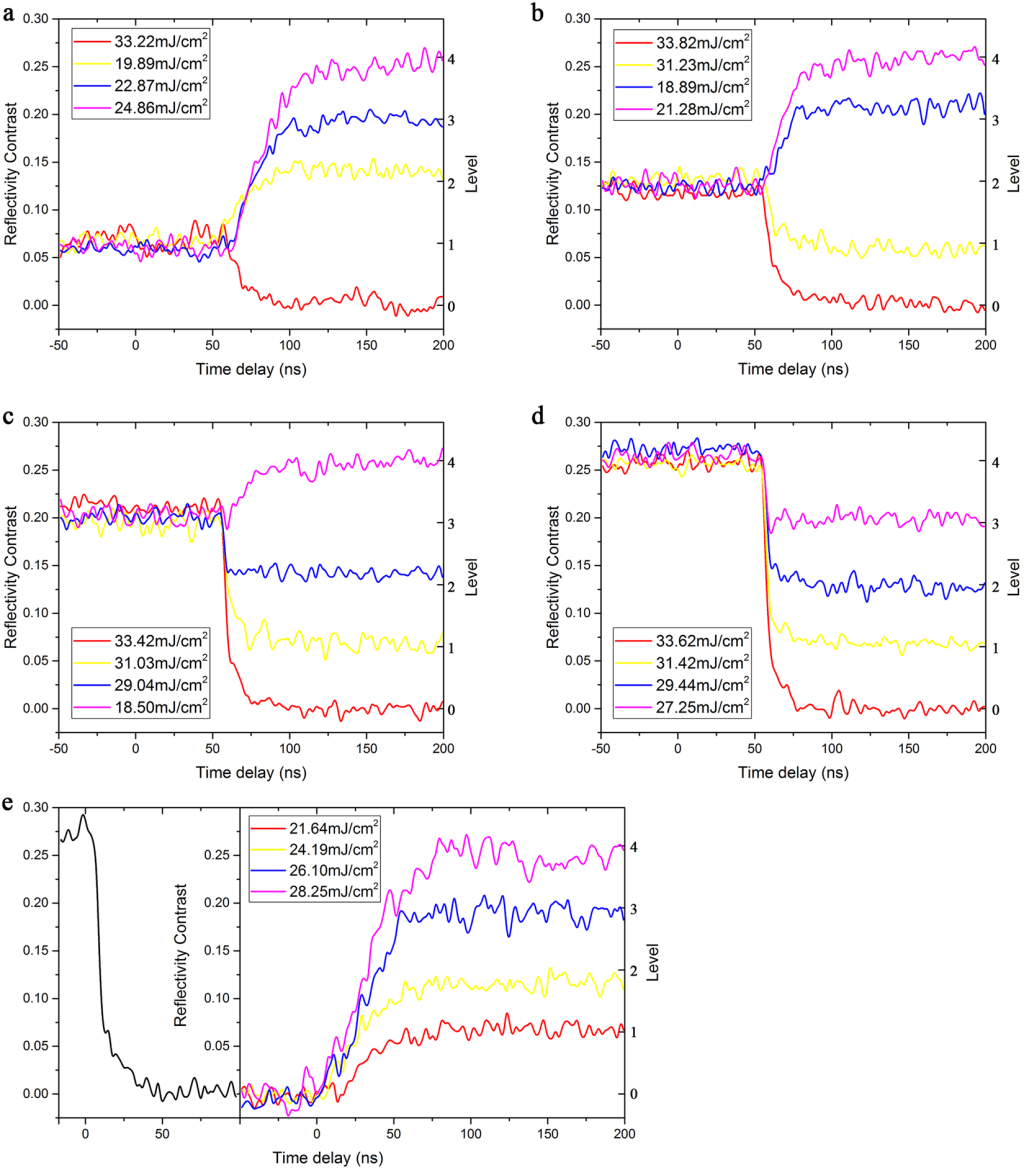
Real-time reflectivity evolution during the transformation between five optical states starting from (**a**) level 1, (**b**) level 2, (**c**) level 3, (**d**) level 4, and (**e**) that of re-establishment of the five optical states (level 0-level 4).

**Figure 3:**
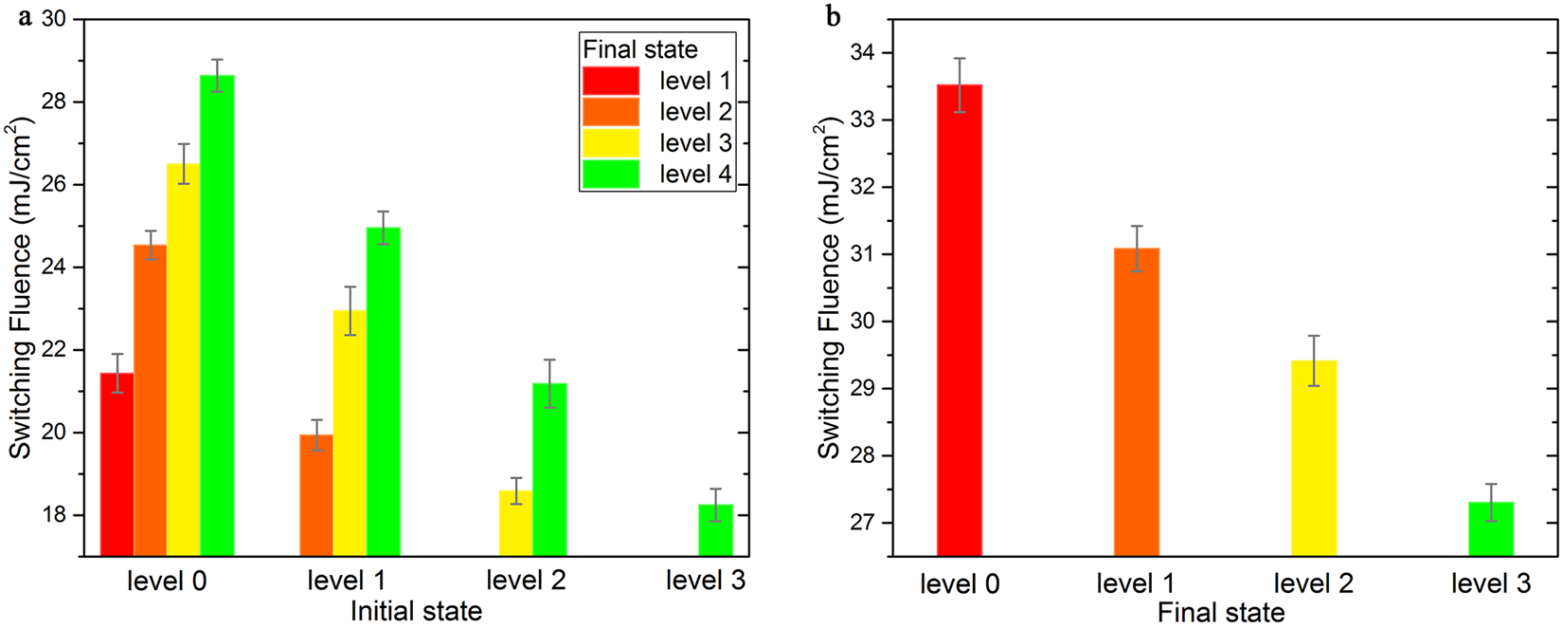
The corresponding laser fluence ranges for the crystallization (**a**) and amorphization (**b**) operations shown in Fig. 2.

Moreover, we will demonstrate the possibility of transformations among the four mentioned states (level 1–level 4) and the initial amorphous phase (level 0). Figure 2 shows the corresponding transient RC change during the transformation. Obviously, by applying single-shot ps-pulses with suitable fluences, each of the established four states and the initial one can be transformed to any of the other four. It should be noted that the four non-zero starting states are achieved from the initial as-deposited state, like that shown in Fig. 1, and the zero starting state (level 0) is formed through amorphization from a partially crystallized state (level 4), like that shown in Fig. 2(e). In other words, the five optical states (level 0–level 4) are re-established. The corresponding laser fluence ranges for the upgoing (crystallization) and downgoing (amorphization) operations are shown in Fig. 3.

From the crystallization dynamics shown in Fig. 2 (the curves in which the final RC is higher than the starting one), we can see that the RC monotonically increases to the final stable state within approximately 50 ns and the recalescence phenomenon cannot be observed in the figures, which is quite different from the multi-stage crystallization process starting from the as-deposited amorphous phase, as shown in Fig. 1. The monotonous increase in the RC from the starting state can also be considered as a solid-state crystallization process. However, it is a little different from the solid-state nucleation process from the pure amorphous state (corresponding to the first increase in the RC shown in Fig. 1). Owing to the existing crystalline grains in the starting states, when the thin films are heated beyond the glass transition point, the generation and growth of crystal nuclei will be promoted, and the crystallization volume will grow quickly according to the nucleation-dominated crystallization nature of GeSbTe. Although this process is more similar to the recalescence-driven crystal growth process, no melting-solidification is needed. Therefore, it is easy to understand why the required energy for crystallization is greatly reduced when we compared the fluence distributions shown in Figs 1 and 2. Moreover, starting states with higher RCs have a greater quantity of existing crystal nuclei and require less energy to increase to the same RC level (crystallization degree).

As for the amorphization process (corresponding to the curves in which the final RC is lower than the starting one), the RC exhibits a typical monotonous decrease. Once the melting point is reached upon the application of ps laser pulses with enough fluences, the GeSbTe thin film loses its crystalline order, and assumes a disordered liquid state. With sufficiently fast heat extraction upon melting, a proper quenching rate for amorphization can be achieved, and thus, the GeSbTe film will be locked in a disordered state^15^. However, when the pulse fluence is relatively low, the undercooling is not enough for full-amorphization, and partial amorphization occurs, which promotes the formation of intermediate states with lower crystallization degrees^24^. It can be noted that the maximal fluence for amorphization (33.82 mJ/cm^2^, Fig. 2b) is close to the minimal fluence for recalescence (surface melting)-involved crystallization (35.81 mJ/cm^2^, Fig. 1). As we know, different starting states (intermediate phases) have different quenching rates under similar fluences, which decide the phase change tendency in the solidification process^14,25^. Interestingly, in the amorphization process, as shown in Fig. 2, no matter the starting state (level), nearly the same quantity of energy will be required to reach a certain final state (level), which confirms that the amorphization process induced by a single-shot ps-laser pulse is independent of the starting state^13^. All the amorphization processes to a certain final state (no matter it is fully or partially amorphous state) seem to have essentially the same process of solidification. Furthermore, the time duration of the amorphization process is sensitive to the pulse fluence, ranging from 10 ns to 30 ns. This behavior can be attributed to an increased melt depth at a higher fluence^13^, which leads to a longer heat extraction time. In fact, the increase in melt depth will not further affect the dynamics of the RC once the optical penetration depth is reached^26^.

### Multi-level coding and recoding operations

**Figure 4:**
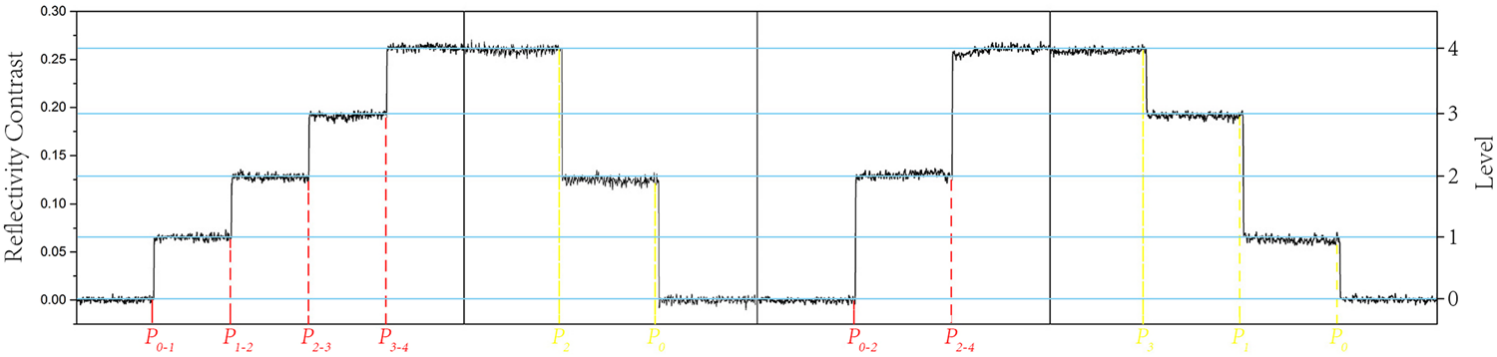
The “sidestep shape” transformation in the RC induced by ps-pulses irradiating on the same spot, where each step represents an optical state (level).

**Figure 5:**
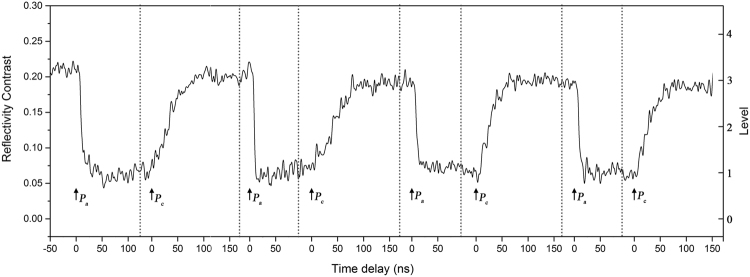
The reversible cycling between level 1 and level 3.

Next, we will discuss the possibility of multi-level cycling (coding-recoding) by applying single-shot ps-laser pulses to the same spot on a Ge_2_Sb_2_Te_5_ thin film. As shown in Fig. 4, every state (level) can be transformed to the other levels repeatedly, which results in a “sidestep shape” in the RC, where each “step” corresponds to a partially crystalline (amorphous) state. In Fig. 4, P_i_ is the inducing pulse of an amorphization process to level i, while P_n−m_ represents the inducing pulse of the crystallization process from level n to level m. Figure 5 shows the reversible cycling between two specified intermediate states (level 1 and level 3). The corresponding fluence for each operation is the same as that shown in Fig. 3. It is demonstrated that not only an individual state (level) can be reached at a high accuracy from both directions, but the repeatability can also be maintained with designed pulse fluences, which is attributed to the excellent reversibility of the GeSbTe material.

### Optical constants, crystalline states and surface morphologies of different optical states

**Figure 6:**
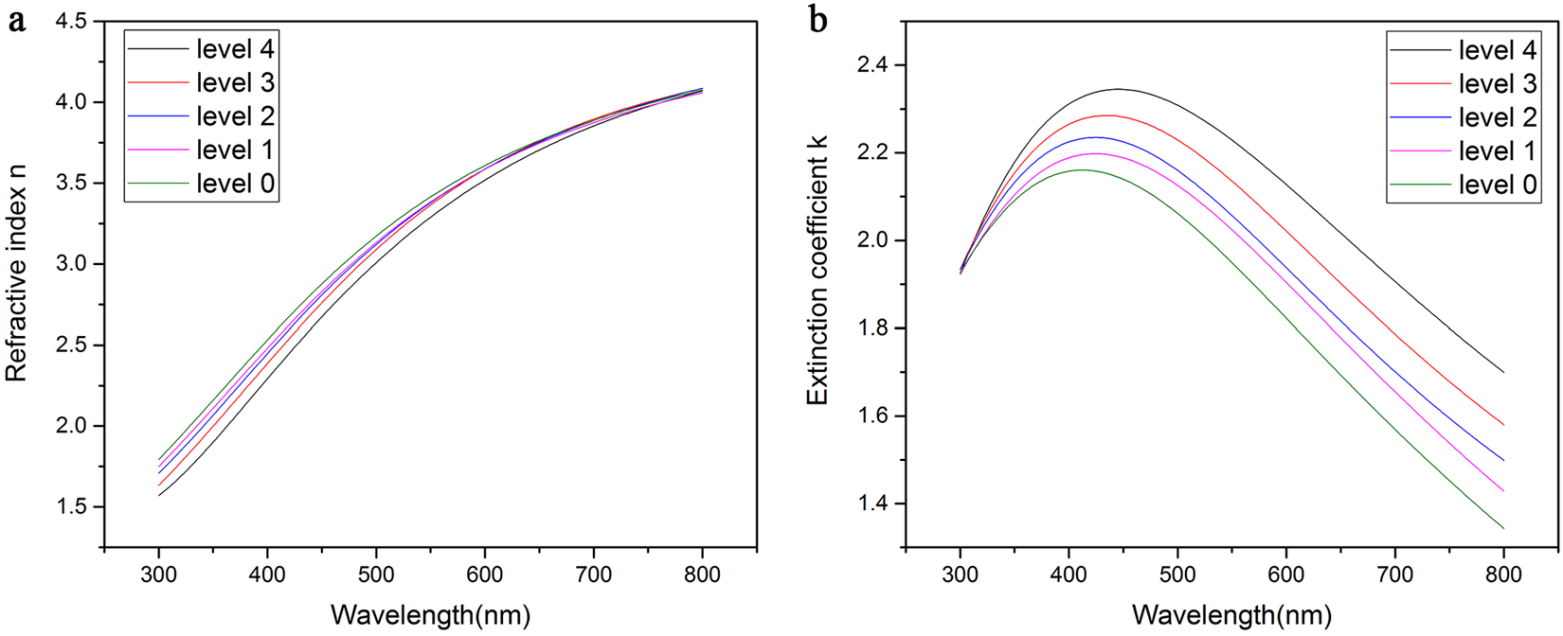
(**a**) Refractive index and (**b**) extinction coefficient of five optical states on the Ge_2_Sb_2_Te_5_ thin film.

As has been established, for any optical meta-material and device, the modulation of optical constants, rather than reflectivity (or the RC) of materials, is more fundamental for the realization of configurable functions^18^. Figure 6 illustrates the optical constants (refractive index and extinction coefficient) of each state in the visible wavelength range. Five clearly distinguishable lines can be seen. With an increase in the crystal proportion of the film (from level 0 to level 4), the refractive index n decreases in the wavelength range of 300–600 nm, while the extinction coefficient k increases in the wavelength range of 350–800 nm, which is in good agreement with the trend of the RC change shown in Figs 1 and 2. The optical constants of all the five optical states are far from that of crystalline state, but very near that of the amorphous state when compared with the results reported in previous studies^27^. It indicates that the above-mentioned five optical states may be amorphous or near-amorphous.

**Figure 7:**
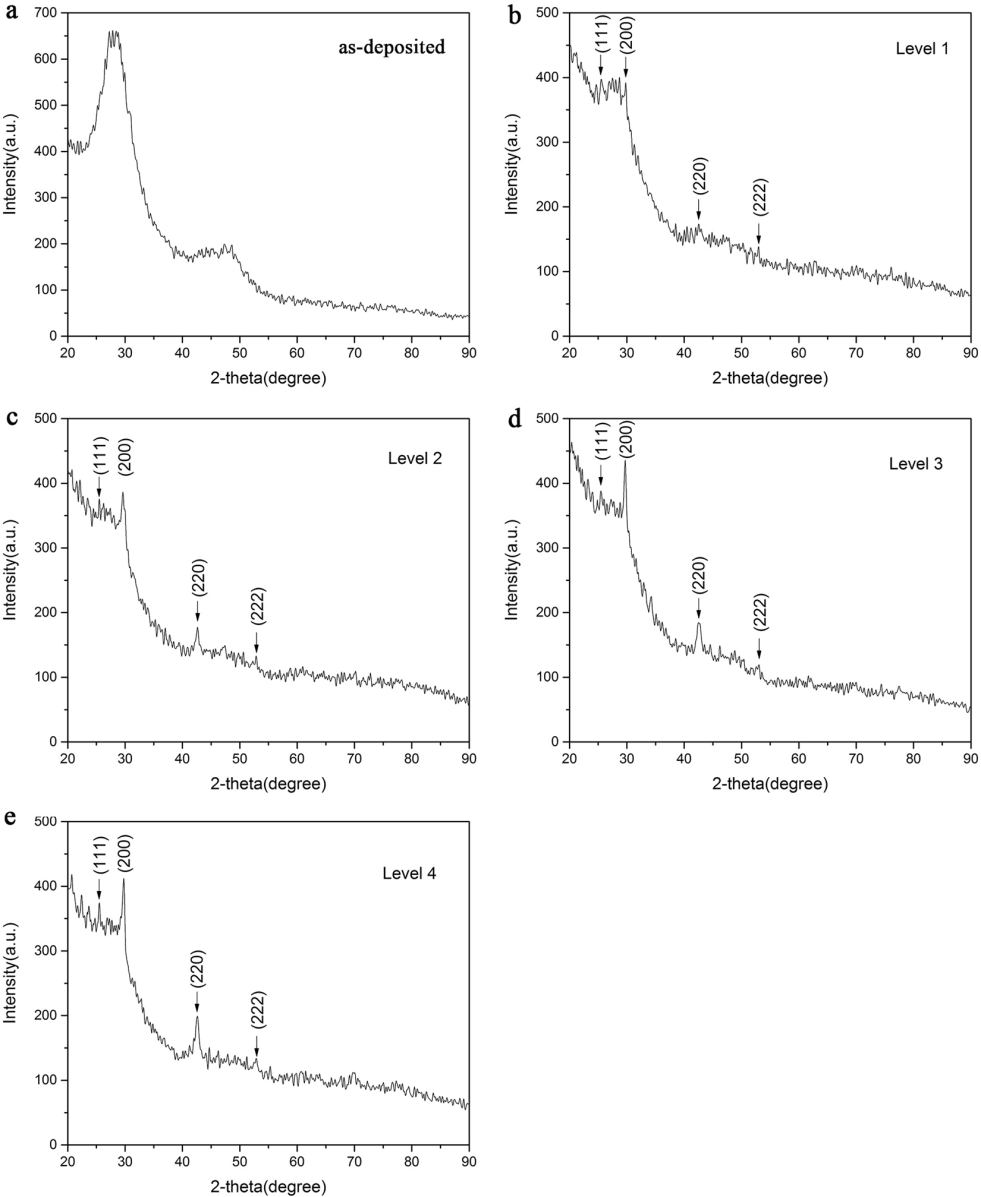
XRD patterns of five optical states on the Ge_2_Sb_2_Te_5_ thin film. (**a**) level 0, (**b**) level 1, (**c**) level 2, (**d**) level 3 and (**e**) level 4.

To confirm the crystalline states of the films at different levels, micro-area XRD analysis is carried out. As shown in Fig. 7, weak crystalline peaks begin to appear after laser irradiation and become stronger gradually with the increased fluences (from level 1 to level 4). However main diffraction features of the as-deposited amorphous state are still maintained for these partially crystallized films.

**Figure 8:**
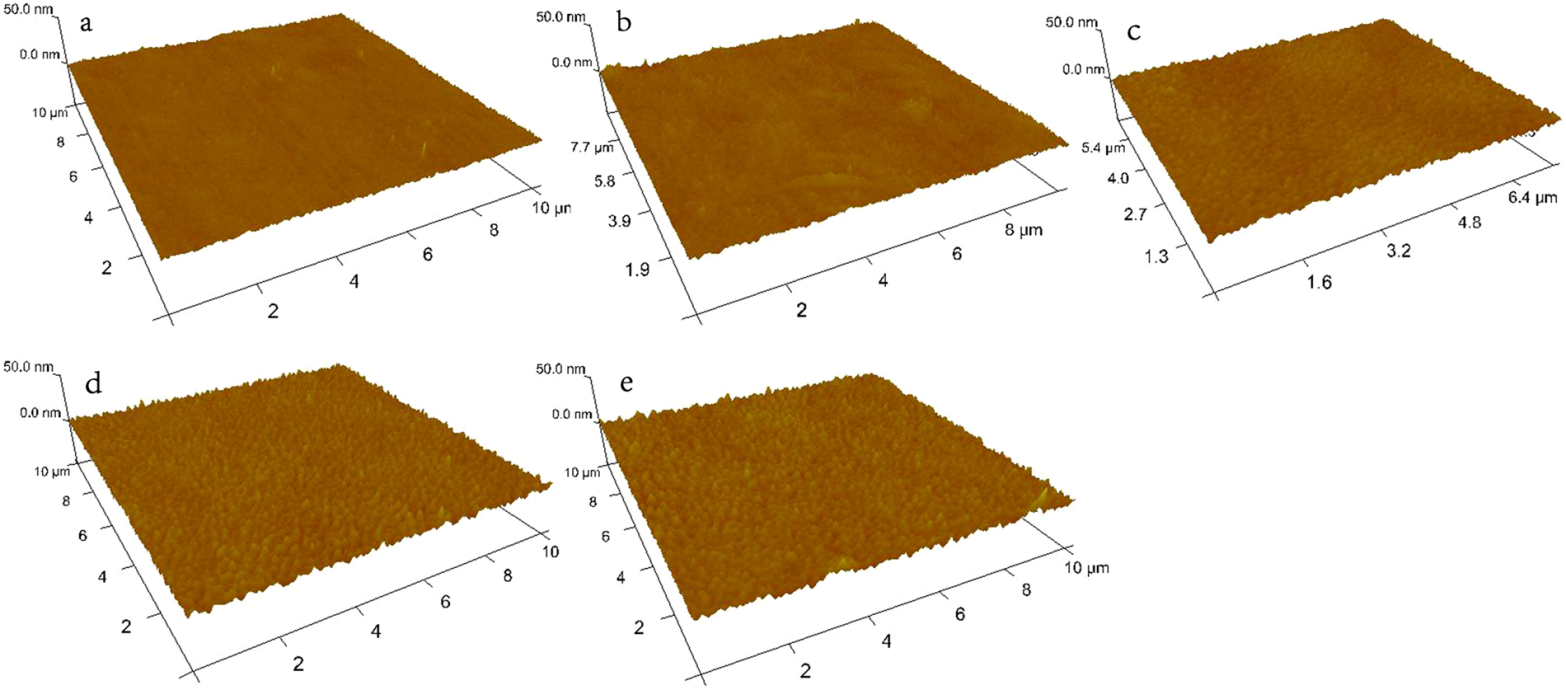
AFM images of the area in (**a**) level 0, (**b**) level 1, (**c**) level 2, (**d**) level 3, and (**e**) level 4. The roughness values are 1.14 nm, 1.70 nm, 2.21 nm, and 2.33 nm, respectively.

Figure 8 shows the AFM images of the as-deposited area and the partially crystallized area of the other four states. With an increase in crystallization degree, the roughness of the film increases gradually. Fine grains gradually appear on the surface of the thin film, and their number increases as well. As analyzed previously, a mixture formed with a partially crystalline compound and its oxide in the process of melting-solidification may explain the presence of progressively more particles^28^. The ultrafast heating time of single-shot ps-pulses and the nucleation-dominated crystallization mechanism of GeSbTe restrict the formation of bigger-sized grains. The maximum surface roughness rate is only a negligible 0.7% for the 170-nm-thick Ge_2_Sb_2_Te_5_ thin films. The maintained smooth surface during ultrafast modulation is favorable for the realization of dynamically configurable functions.

Those microstructural features agree well with the results of optical measurements.

In summary, the ultrafast reversible phase transition processes among different amorphous or near-amorphous intermediate states are reported for the first time to the best of our knowledge. We demonstrate that by applying single-shot picosecond-pulses with suitable fluences, each of the established five states (level 0–4) can be transformed to any of the other four. We demonstrate the reversible switching between two intermediate states by applying single-shot ps-pulses with suitable fluences. We find several interesting characteristics of the classic phase change material Ge_2_Sb_2_Te_5_ during these ultrafast coding-recoding processes. In the amorphization process between two intermediate states, no matter the starting (initial) state, nearly the same quantity of energy will be required to reach a certain final state. We explain these observations based upon a combination of optical and microstructural analysises. These results may be constructive for the deeper understanding of the ultrafast multi-level phase-transition mechanics of chalcogenides, and lay a foundation for the further design and application of dynamically reconfigurable metasurfaces^29–32^ and optical/photonic devices^33–42^.

## Methods

### Thin film preparation

Ge_2_Sb_2_Te_5_ thin films with a thickness of 170 nm were deposited on K9 glass substrates at room temperature by a radio frequency magnetron controlling sputtering system, where the background pressure was approximately 7.5 × 10^−4^ Pa, the sputtering power was 20 W and the sputtering pressure was approximately 0.5 Pa. The thickness of the films was measured using a surface profiler (Tencor AlphastepD100).

### Multi-level coding and recoding operations

The establishment and transformation processes of different optical states were realized and observed using a self-developed real-time pump-probe optical system^14^. A picosecond mode-locked Nd:YAG laser with a wavelength of ~1064 nm and a pulse duration of ~30 ps was used as the pump source. The laser beam was focused by a convex lens onto the front surface of a Ge_2_Sb_2_Te_2_ thin film with a spot diameter of approximately 1 mm. The probe light beam was from a continuous-wave semiconductor laser with a wavelength of 650 nm and was incident at an angle of ~45° at the center of the irradiated area with a spot diameter of 0.3 mm. The reflecting beam was collected and measured by a high-speed silicon avalanche photodiode and a digital phosphor oscilloscope. The total time resolution of the pump-probe system was approximately 2 ns.

### Structural and optical characterizations

The surface morphology of the irradiated spot was observed using an atomic force microscope (Veeco MultiMode V). The optical constant of the Ge_2_Sb_2_Te_2_ thin film at different optical states was measured using a spectral ellipsometer (Sopra GES5E UV/Vis/IR). Crystal structure analysis for the as-deposited films and laser-irradiated spots was carried out by micro-area X-ray diffraction (Rigaku D/max-2550).
